# Incessant Focal Atrial Tachycardia Originating From the Right Inferior Pulmonary Vein With Extremely Short Cycle Length and Variable Exit Block

**DOI:** 10.1155/cric/9933004

**Published:** 2026-05-23

**Authors:** Hidemori Hayashi, Gaku Sekita, Tohru Minamino

**Affiliations:** ^1^ Department of Cardiovascular Biology and Medicine, Juntendo University Graduate School of Medicine, Tokyo, Japan, juntendo.ac.jp

**Keywords:** catheter ablation, focal atrial tachycardia, right inferior pulmonary vein

## Abstract

The underlying mechanism of focal atrial tachycardia (AT) is usually considered to be abnormal automaticity, typically with a cycle length (CL) exceeding 120 ms. We present a case of a 29‐year‐old female with recurrent symptomatic palpitations. She was referred for electrophysiologic study and catheter ablation. Twelve‐lead ECG showed a sinus rhythm with incessant atrial ectopic beats and occasional aberrant conduction. P‐wave morphology during ectopic revealed positive deflections in Leads I, II, aVL, aVF, and V1–V6, suggesting a left‐sided origin. Intracardiac recordings obtained using a circular multipolar mapping catheter demonstrated incessant, repetitive, regular spike potentials with an extremely short CL at the posterior aspect of the right inferior pulmonary vein (RIPV). The tachycardia CL within the RIPV was approximately 70 ms and was conducted to the left atrium (LA) with variable exit block ranging from 2:1 to 4:1 conduction. Successful ablation was performed at the earliest activation and shortest CL site outside the RIPV with exit block from the RIPV to LA. The mechanism for this tachycardia is uncertain, but the very short CL suggests that a localized spiral wave re‐entry may be involved.

## 1. Introduction

Focal atrial tachycardia (AT) is the least common form of supraventricular tachycardia. The typical underlying mechanism is enhanced automaticity. Anatomic clustering is well described, and reported tachycardia cycle lengths (TCLs) generally exceed 120 ms. Herein, we present a case of successful ablation for incessant focal AT originating from the right inferior pulmonary vein (RIPV) with extremely short cycle length and variable exit block.

## 2. Case Report

**Figure 1:**
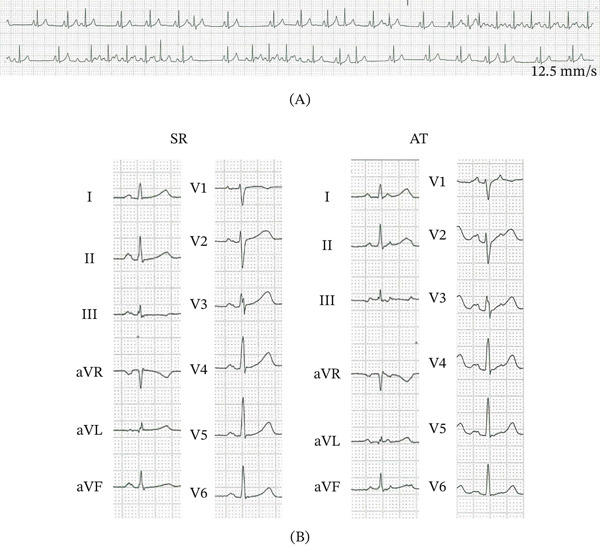
(A) The rhythm strip shows atrial ectopic beats and nonsustained atrial tachycardia mimicking atrial fibrillation. (B) Twelve‐lead ECG during a sinus rhythm and atrial tachycardia. P‐wave morphology on AT demonstrates positive deflection in Leads I, II, aVL, aVF, and V1–V6, and the PP interval was approximately 0.3 s. SR = sinus rhythm; AT = atrial tachycardia.

A 29‐year‐old female with incessant palpitations refractory to multiple antiarrhythmic drugs including beta‐blockers and a Class Ic antiarrhythmic agent was referred for electrophysiological study (EPS) and catheter ablation. Transthoracic echocardiography revealed normal left ventricular function without left atrial dilatation. Twelve‐lead ECG on admission showed a sinus rhythm with incessant atrial ectopic beats and nonsustained AT with irregular ventricular response mimicking atrial fibrillation (AF) (Figure 1A). P‐wave morphology was monomorphic during atrial ectopic and AT, featuring positive deflection in Leads I, II, aVL, aVF, and V1–V6, consistent with a left‐sided focus (Figure 1B).

**Figure 2:**
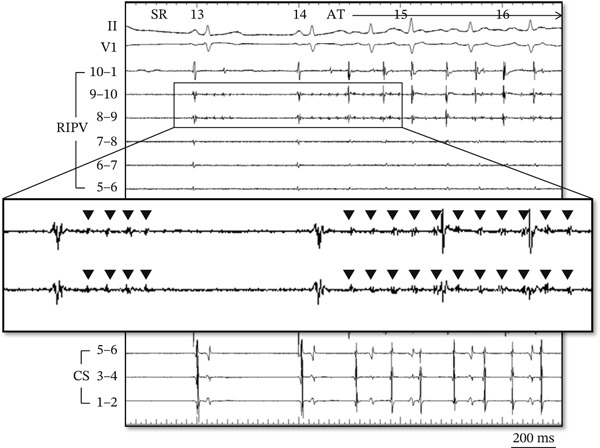
Intracardiac electrograms at the spontaneous initiation of AT with repetitive regular potentials (black down‐pointing triangle) in the RIPV. Nonsustained firing during the first sinus beat is not conducted to the adjacent LA. AT is then induced by the next rapid burst from the RIPV with a TCL of approximately 70 ms. SR = sinus rhythm; AT = atrial tachycardia; RIPV = right inferior pulmonary vein; LA = left atrium; TCL = tachycardia cycle length; CS = coronary sinus.

**Figure 3:**
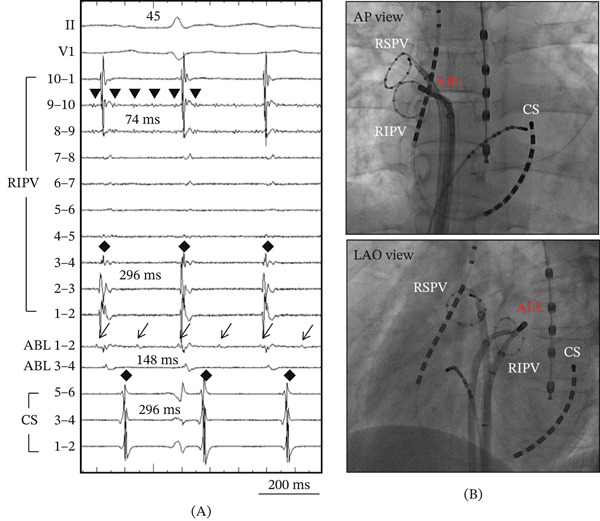
(A) Intracardiac electrograms demonstrate variable exit block during AT activation to the ablation catheter (ABL) site with 2:1 (arrow), then RIPV and CS with 4:1 conduction block (black diamond). ABL was located at the posterior aspect of the RIPV, and RF ablation was performed at this site, resulting in successful termination of AT. (B) Fluoroscopic view of the successful ablation site. RSPV = right superior pulmonary vein; RIPV = right inferior pulmonary vein; CS = coronary sinus; AP = anteroposterior; LAO = left anterior oblique.

At baseline EPS, the patient was spontaneously in clinical tachycardia. During detailed activation mapping of the left atrium (LA) and pulmonary veins, incessant repetitive regular potentials were noted on the circular mapping catheter at the posterior roof of the RIPV within 1 cm of the RIPV ostium. The potentials were discrete, focal, fragmented, and very low amplitude (Figure 2). The TCL at the localized area within the RIPV was approximately 70 ms. Conduction to the adjacent LA and subsequent spread to the periphery occurred with a variable (2:1–4:1) exit block (Figure 3A). Although the TCL in the RIPV was extremely short, conduction to the LA was organized without induction of AF throughout the case. Radiofrequency ablation was performed using a 3.5‐mm open‐irrigated tip ablation catheter at the posterior aspect of RIPV antrum (Figure 3B) at a site of 2:1 conduction close to the focal origin, resulting in immediate exit block from the RIPV to LA on the first application. Energy was delivered at 30–35 W with a maximum temperature limit of 43°C for 20–30 s at each site. Additional ablation of the focal origin abolished all remaining local activity. No further AT was observed or inducible using atrial programmed stimulation despite repeated attempts and use of varying doses of isoproterenol. There were no procedural complications. The patient remained completely free of palpitations for more than 12 months after the ablation procedure, and no recurrence of atrial arrhythmias was documented on serial outpatient ECGs or 24‐h Holter monitoring.

## 3. Discussion

To the best of our knowledge, this is the first case report of a focal AT with extremely short cycle length (< 100 ms) and varying degree of intra‐atrial conduction block.

Focal AT is an uncommon form of supraventricular tachycardia, accounting for approximately 10%–15% of all cases, and often occurs in the absence of structural heart disease [1]. Despite being considered a benign tachycardia, up to 30% of patients with incessant AT eventually develop a tachycardia‐mediated cardiomyopathy [2]. Potential mechanisms underlying focal AT include abnormal automaticity, triggered activity, or localized re‐entry. Catheter ablation of focal AT is a highly effective and feasible treatment with most studies reporting acute and long‐term success rates in excess of 85%–90% and a low incidence of major complications [1, 3].

The foci of AT are distributed to characteristic anatomical sites throughout the atria [4]. Approximately 75% occur in the right atrium, and common sites of origin include the crista terminalis, the tricuspid annulus, the ostium of the coronary sinus, and the para‐Hisian region [5]. Left atrial foci are often located at the ostium of pulmonary veins and along the mitral annulus [4]. The TCL of focal AT has been reported to be approximately 350 ± 100 ms, whereas for pulmonary vein tachycardia in patients with AF, TCL is reported at 130 ± 30 ms [3, 6]. Sanders et al. reported the mechanism of focal AT using a high‐density contact mapping catheter. Among a total of 27 ATs, a localized focus was observed at the site of origin in 19 ATs (70%), whereas in 8 (30%), localized re‐entry was evidenced by 95.2*%* ± 4.5*%* of TCL recorded within the mapping field indicating an “anatomical re‐entry mechanism” in the small area. They additionally described that the localized re‐entry group had a shorter TCL compared with focal origin group (median TCL [range; millisecond]: 246 ms [190–306] vs. 424 ms [210–550], respectively, *p* = 0.009), and most of the cases (7/8, 88%) in localized re‐entry group had a history of AF ablation and evidence of slowed conduction such as contiguous scar or double potentials [7].

In the present case, although electroanatomical mapping using the CARTO system was attempted, detailed activation and voltage mapping during AT was not feasible due to the extremely short cycle length and the incessant form of the tachycardia with variable exit block. Furthermore, pacing maneuvers aimed at demonstrating a re‐entrant mechanism could not be performed because reliable pacing capture and interpretation were not possible. Therefore, although the exact mechanism could not be definitively determined in our case, several electrophysiological characteristics argue against simple focal automaticity or triggered activity. These include the extremely short and stable TCL (approximately 70–80 ms), the presence of a variable exit block from the RIPV to LA, and the immediate termination of tachycardia by focal ablation with creation of an exit block. Given these findings, a localized functional re‐entrant mechanism confined within the pulmonary vein musculature, in which a single spiral wave is generated by an impulse occurring in the wake of a propagating wave during the vulnerable period, appears to be a plausible explanation. The absence of fractionated or double potentials in the adjacent LA and the lack of prior AF or atrial scarring further support a functional rather than anatomical re‐entry.

## 4. Conclusions

We describe a rare case of incessant focal AT originating from the RIPV with an extremely short cycle length and variable exit block. Although the precise mechanism remains uncertain, this unique electrophysiological phenotype suggests that a localized spiral wave re‐entry may be involved.

## Author Contributions

Hidemori Hayashi: conceptualization, investigation, writing—original draft, and writing—review and editing; Gaku Sekita: investigation and writing—review and editing; Tohru Minamino: supervision and writing—review and editing.

## Funding

No funding was received for this manuscript.

## Consent

Informed consent was obtained from the patient who was described in the case report. The participant has consented to the submission of the case report to the journal.

## Conflicts of Interest

The authors declare no conflicts of interest.

## Data Availability

The data that support the findings of this study are available from the corresponding author upon reasonable request.
